# Clinical thresholds in pain-related facial activity linked to differences in cortical network activation in neonates

**DOI:** 10.1097/j.pain.0000000000002798

**Published:** 2022-10-27

**Authors:** Oana Bucsea, Mohammed Rupawala, Ilana Shiff, Xiaogang Wang, Judith Meek, Maria Fitzgerald, Lorenzo Fabrizi, Rebecca Pillai Riddell, Laura Jones

**Affiliations:** a Psychology, Faculty of Health, York University, Toronto, ON, Canada; b Department of Neuroscience, Physiology and Pharmacology, University College London, London, United Kingdom; c Department of Mathematics and Statistics, York University, Toronto, ON, Canada; d University College London Hospital, London, United Kingdom; e Psychiatry, Hospital for Sick Children, Toronto, ON, Canada; f Psychiatry, University of Toronto, Toronto, ON, Canada

**Keywords:** Infant pain, Pain-related facial activity, Nociceptive processing, Brain-behavior relationships

## Abstract

In neonates, a noxious stimulus elicits pain-related facial expression changes and distinct brain activity as measured by electroencephalography, but past research has revealed an inconsistent relationship between these responses. Facial activity is the most commonly used index of neonatal pain in clinical settings, with clinical thresholds determining if analgesia should be provided; however, we do not know if these thresholds are associated with differences in how the neonatal brain processes a noxious stimulus. The objective of this study was to examine whether subclinical vs clinically significant levels of pain-related facial activity are related to differences in the pattern of nociceptive brain activity in preterm and term neonates. We recorded whole-head electroencephalography and video in 78 neonates (0-14 days postnatal age) after a clinically required heel lance. Using an optimal constellation of Neonatal Facial Coding System actions (brow bulge, eye squeeze, and nasolabial furrow), we compared the serial network engagement (microstates) between neonates with and without clinically significant pain behaviour. Results revealed a sequence of nociceptive cortical network activation that was independent of pain-related behavior; however, a separate but interleaved sequence of early activity was related to the magnitude of the immediate behavioural response. Importantly, the degree of pain-related behavior is related to how the brain processes a stimulus and not simply the degree of cortical activation. This suggests that neonates who exhibit clinically significant pain behaviours process the stimulus differently and that neonatal pain-related behaviours reflect just a portion of the overall cortical pain response.

## Abstract 2

Supplemental Digital Content is Available in the Text.

Subclinical and clinically significant pain-related facial expressions in neonates are related to distinct patterns of early cortical network activation after a noxious stimulus.

## 1. Introduction

In neonates, a noxious stimulus elicits pain-related changes in facial expression and distinct brain activity as measured by electroencephalography (EEG).^1,20,28,41^ These responses are typically considered directly related: greater amplitude of the EEG response at the scalp vertex corresponds to stronger or more prolonged facial expressions.^24,27^ However, this relationship can be disrupted by factors such as stress and sucrose administration.^27,42^ Moreover, many infants exhibit no or subclinically significant behaviours, despite a significant cortical response.^26,40^ This is important because pain-related behaviour scales are a significant component of many measures of neonatal pain in clinical settings, for which clinical thresholds have been imposed that determine if analgesia should be provided.^4^ It is not known if these clinical thresholds are related to differences in how the neonatal brain is processing the noxious stimulus.

Although the afferent input from the spinal cord is the same for both cortical and facial responses, their pathways diverge at the brainstem. Facial expressions are initiated by activation of the facial nerves in the pons,^6,37^ whereas the cortex is activated by ascending projections through the brainstem and thalamus.^5,7^ Initial pain-related changes in facial expression communicate distress to bring about a caregiver response.^56^ Initial facial expressions are considered reflexive behaviours, as immature neonates are incapable of volitional facial control, which requires complex communication between sensorimotor, frontal, and limbic cortices.^21^ Cortical activation, on the other hand, is considered to underpin awareness of the stimulus.^5^

Processing of a noxious stimulus requires the parallel and sequential activation of a distributed network of brain regions,^5,61^ with different regions subserving distinct cortical processes that are engaged hierarchically.^3,14,50,51^ While the previous assessment of the relationship between behaviour and cortical activity has been limited to the nociceptive event–related potential (N3P3) at a single electrode (vertex) and time point (500-700 ms poststimulus), the use of global event–related topographical analysis (microstates) can capture this complex serial activation,^38,55^ allowing for the examination of potentially distinct cortical functions related to subclinical and clinical facial expression changes. An important consideration in this relationship is the rapid development of the neonatal brain over the final trimester of gestation,^53^ with different brain regions and networks developing at different rates.^11,18,19^ This development is accompanied by the refinement of behavioural responses^12,13^ and the continuous evolution of the sequential cortical network engagement following a painful procedure.^55^

The aim of this study is to determine if subclinical or clinical pain behaviours in late preterm and term neonates are related to differences in nociceptive processing in the brain. To do this, we compare the serial network engagement (microstates) from whole-head EEG recordings between neonates with and without clinically significant pain behaviour after a clinically required blood test. We hypothesise that clinically significant facial expression changes are related to the engagement of distinct networks in the cortex, rather than the power of the overall brain response. The results provide insight into the emergence of an integrated model of brain and behaviour in the newborn infant pain response.

## 2. Methods

### 2.1. Participants

The current study draws on an archival sample of 78 neonates (41 late preterm and 37 full-term neonates [Table 1]), ranging from 0 to 14 days postnatal age recruited from the postnatal, special care, or intensive care wards at the Elizabeth Garett Anderson Obstetric Wing, University College London Hospital (UCLH) in London, England in the period of June 2010 to May 2018. Infants who had grade 4 hypoxic ischaemic encephalopathy, periventricular haemorrhage, >grade 2 intraventricular haemorrhage, trisomy 21, intrauterine growth restriction, or were prescribed opioids at the time of the study were not included in this analysis. Approval for data collection was obtained from the National Health Service Health Research Authority (London—Surrey Borders). Approval for the current analyses was obtained from the York University Ethics Review Board.

**Table 1:** Participant demographics.

	Preterm (N = 41)	Full term (N = 37)	*t*(50-76)/χ^2^(1)	*P*
GA* (wk)	35.24 (1.03)	38.91 (1.18)	−14.66	0.00
PNA† (d)	6.10 (3.91)	5.03 (2.87)	1.37	0.18
Females	24 (58.54)	12 (32.43)	5.33	0.02
Apgar score at 5 min‡	9.20 (0.99)	9.27 (1.19)	−0.28	0.78
Birth weight (g)	2111.37 (374.53)	3066.41 (610.63)	−8.42	0.00
Skin-breaking procedures§	17.83 (9.08)	11.91 (7.82)	2.48	0.02

Counts (%) provided for sex ratios. M (SD) provided for all other variables. χ^2^ statistics provided for sex ratios; *t* test values provided for all other variables.

* GA refers to gestational age—number of weeks from the first day of the mother's last menstrual cycle to birth.

† PNA refers to postnatal age—number of days since birth.

‡ Scores are on a scale of 0 to 10, with higher scores indicative of higher newborn well-being.

§ Total number of skin-breaking procedures neonates was exposed to before participating in the study.

### 2.2. Experimental design

Brain activity (up to 18 electrode EEG) and facial expressions (video) were recorded following a single clinically required noxious heel lance at bedside in the neonatal unit. Informed written parental consent was obtained before the study. Analyses were conducted separately for preterm (33-36 weeks gestational age) and term (37-40 weeks gestational age) neonates due to past literature which has uncovered differences in the degree of facial expression changes^13,57^ and the engagement of distinct cortical networks (microstates^55^) following a heel lance procedure.

### 2.3. Noxious procedure

The noxious event was a clinically required heel lance performed by the same trained research nurse. Standard hospital practice was followed during all heel lances. The heel was cleaned with sterile water, after which the nurse placed the lancet on the infants' skin for 30 seconds without releasing it, to obtain baseline data free of other stimulation. The release of the lance was time locked to the ongoing EEG recording using an accelerometer mounted onto the lancet.^58^ After the blade was released, the nurse did not squeeze the infants' foot for another 30 seconds to ensure that the post lance data were again free of other stimulation. Parents were allowed to comfort their infants as desired, which is consistent with the hospital protocol encouraging parent-led pain management techniques during painful procedures. More information regarding this study's methodology is described in Jones et al. ^28^

### 2.4. Pain-related facial actions

#### 2.4.1. Coding

Video footage of neonates' pain-related facial actions was coded using the 7-item version of the Neonatal Facial Coding System (NFCS) (brow bulge, eye squeeze, nasolabial furrow, open lips, vertical stretch mouth, horizontal stretch mouth, and taut tongue).^13,22^ Neonatal Facial Coding System has demonstrated good psychometric properties, such as reliability, convergent validity, and construct validity.^31,44,44^

As various constellations of these facial actions have been used in the study of neonatal pain, we first sought to compare commonly used constellations to determine the one that optimally captures the full range of pain-related behavioural expressions in term and preterm neonates. The constellations explored were as follows: (1) 3-item NFCS score (NFCS-3) consisting of eye squeeze, vertical stretch mouth, and horizontal stretch mouth, based on work with older term infants,^17^ (2) 3-item NFCS score consistent with the facial actions included in the Premature Infant Pain Profile-Revised (NFCS-P-3; brow bulge, eye squeeze, and nasolabial furrow) and one of the most highly used infant pain measures,^45^ and (3) 7-item NFCS score (NFCS-7) consisting of all facial actions.^1^ Higher scores on all constellations are indicative of more pain-related facial actions and presumably greater pain-related distress.

Each facial action was coded second by second as either 0 (not present) or 1 (present) and summed over all facial actions (7 or 3) over 2 epochs: (1) 10 seconds immediately prelance (baseline) and (2) 10 seconds immediately postlance (reactivity). Therefore, the maximum score per epoch was either 70 (7 facial action constellation) or 30 (3 facial action constellation). Two trained coders who were blinded to study hypotheses, neonate status (late preterm vs full term), and the microstate analyses coded all the data. Thirty percent of the data was coded for interrater reliability. Ongoing reliability throughout the coding process was also examined to prevent coder drift. Intraclass correlations (ICCs), a measure of interrater reliability, ranged from 0.90 to 1.

#### 2.4.2. Missing data management

Coding NFCS in a hospital setting in the context of acute painful procedures is challenging as the view of the infant face on video is often obstructed due to infant movement or medical equipment. To prevent the systematic bias inherent in excluding infants who moved during the procedure or those who required more intensive medical care, missing data were managed using 3 previously established procedures,^1,36^ which allowed coders to make conservative judgements about missing facial actions. First, if half of the infant's face could be seen on video and all facial actions could be coded from the visible half of the face, then the items would be coded based on the assumption of facial symmetry. This method was used in 15% of trials. Second, if facial actions were actually obstructed, a blinded coder reviewed the video and determined the cause of missing data (eg, infant turned the face away from the camera). Then, the coder examined whether 2 other commonly used pain-related distress behaviours (eg, cry and body movements)^46^ remained constant while the infants' face was obstructed. The assumption was made that if cry and body movements remained constant, it is highly probable that facial actions also remained constant during that time, and the missing value was estimated as the closest preceding value available. To use this constancy method, 3 conditions had to be satisfied: (1) facial activity had to be available and codable for at least 60% of the 10 seconds epoch, (2) cry and body movements had to be available and remain constant throughout the 10 seconds epoch, and (3) the coder did not have any other reason to believe facial activity did not remain constant when obstructed. Across the 7 facial actions, 5% of data was estimated using this constancy rule. Third, if the first criteria of the constancy rule was not met (eg, due to a facial action being available for less than 60% of the 10 seconds epoch), but the other pain-related distress behaviours (eg, cry and body movements) remained constant, data were prorated if at least 60% of the overall coding across all the actions was available. Consensus had to be reached among at least 2 coders that the constancy rule could not be applied before scores were prorated. This procedure was used with 5% of participants with 10% to 33% missing data. Finally, if less than 60% of data was available and proration was not feasible, the participant was excluded from the sample. Overall, only one participant had a missing NFCS-P-3 score and was thus excluded from the analyses.

#### 2.4.3. Selecting the optimal constellation of facial actions

Before exploring the relationship with brain activity, we determined the optimal constellation of facial actions, which was considered to be the one that captured the most variability (complete range of possible facial action scores) in pain-related behavioural levels. We did this by assessing (1) the occurrence of each of the 7 individual facial actions across infants, (2) the magnitude of the overall facial expression score using each of the 3 constellations, and (3) the interindividual variability resulting from the 3 constellations.

An examination of Shapiro–Wilk normality tests, Q-Q plots, and frequency distributions revealed that the 3 constellations of pain-related facial actions (NFCS-7, NFCS-3, and NFCS-P-3) were not normally distributed; therefore, nonparametric tests were conducted to compare the occurrence, magnitude, and interindividual variability of these scores. Mann–Whitney *U* tests were used to compare facial action scores between preterm and full term neonates. Frequency distributions, Friedman analysis of variance, and Wilcoxon signed-rank tests were used to examine differences in patterns of pain-related facial activity separately in preterm and term neonates. Neonates were then split into groups of subclinical and clinically significant facial activity score, using the score derived from the optimal constellation, and their brain activity compared.

### 2.5. Electroencephalography

#### 2.5.1. Recording

Electroencephalography responses time locked to the heel lance were recorded from up to 18 disposable electrodes (Ag/AgCl cup electrodes). Electrodes were placed on the scalp according to the international 10/20 electrode placement system covering the primary visual (O1 and O2), primary auditory (T7 and T8), association (F7, F3, F4, F8, P7, P8, TP9, and TP10), and somatosensory (C3, Cz, C4, CP3, CPz, and CP4) scalp areas. A reference electrode was placed at Fz and the ground electrode at FC1/2, depending on the positioning of the infant during the procedure. Electrode impedance was minimized by rubbing the scalp with a prepping gel (NuPrep, Weaver & Co, Aurora, CO) and then applying the electrodes using a conductive paste (10/20 Weaver & Co). A soft bonnet was placed on the scalp to secure the electrodes. The Neuroscan SynAmps2 recording system was used to record the activity from DC to 500 Hz. Signals were digitised using a sampling rate of 2 kHz and resolution of 24 bit. All EEG data were examined by a trained neurophysiologist, and no EEG abnormalities were observed.

#### 2.5.2. Preprocessing

Electroencephalography data were preprocessed in EEGLAB and custom MATLAB scripts. Second-order bidirectional Butterworth bandpass (1-30 Hz) and notch (48-52 Hz) filters were applied to the raw data. Remaining artifacts (eg, high amplitude activity or ECG signals) were removed using independent component analysis in EEGLAB.^15^ Artifactual independent components were selected manually using the spatial maps and frequency content of the components. If channels were not recorded or could not be denoised, spherical interpolation was used to estimate them (average # estimated channels = 0.4, range 0-4). Finally, data was rereferenced to the common average and epoched to 0.5 seconds before and 1 second after the lance. All preprocessing was conducted by the same experienced researcher.

#### 2.5.3. Comparison between term and preterm cortical activity

TANOVA testing^52^ was used to determine if cortical activation was different between term and preterm neonates. This test provides an index of how different (dissimilarity index [DISS]) 2 scalp field topographies are, which we explored for each timepoint in the baseline and poststimulus period (−500 to 1000 ms). The DISS can range from 0 (no difference) to 2 (completely reversed in polarity). Timepoints with significantly different topographies were determined with bootstrapping. A nonparametric null distribution was obtained by shuffling subjects across the 2 age groups and recalculating the DISS (5000 iterations). If the true DISS, at a given timepoint, was larger than the 95th percentile (right-tailed, *P* < 0.05) of the nonparametric null distribution, the topographies were considered significantly different. To account for multiple comparisons, only time periods which were continuously significant for 5% of the overall test window (−500 to 1000 ms) were considered significant.

#### 2.5.4. Scalp field analysis (microstates)

We investigated the difference in the total power and patterns of cortical activity (microstates) between the 2 facial activity groups. Microstate analysis was used in place of the traditional event-related potential (ERP) analysis at the vertex electrode, due to the additional information which can be obtained.^39^ Importantly, using information from a full scalp electrode array can detect differences which are otherwise missed using ERP analysis. Microstate analysis was conducted using Ragu^23^ and custom-written MATLAB scripts. For a full description of the analysis, see Rupawala et al. ^38^

First, for each group average, we calculated the global field power (GFP, standard deviation of the cortical activity across all electrodes at every timepoint). The GFP represents the overall amount of activity at each time point considering all recording electrodes simultaneously. This results in a reference-independent descriptor of the potential field sampled at the scalp. Next, we used a topographic consistency test (TCT) to identify spatially consistent ERP maps after the heel lance.^23^ The time period examined commenced at 200 milliseconds before the stimulus trigger (−200 to 1000 ms) due to a period of uncertainty as to the exact release of the lance.^58^ The TCT assesses the similarity of the topography of scalp potentials within groups. Data at timepoints that have a significantly consistent topography across subjects reflect event-related activation, whereas random fluctuations in cortical activity, unrelated to the stimulus or not specific to the group, are unlikely to be consistent across participants at a given latency.^23^ Significantly consistent timepoints were determined with bootstrapping. A nonparametric null distribution was obtained by shuffling data across electrodes for each subject (altering the consistency at each channel across subjects) and recalculating the GFP of the average (5000 iterations). If the true GFP of the average, at a given latency, was larger than the 95th percentile (right-tailed, *P* < 0.05) of the nonparametric null distribution, the topography was considered consistent.

Data at all consistent timepoints across all subjects and groups were pooled, from which microstates were identified using hierarchical clustering of data with similar topographies. To determine the optimal number of clusters (ie, microstates), a random 50% of subjects were used to identify clusters, and the remaining 50% were used to check the amount of signal explained by those clusters (cross-validation, 100 iterations). Once the optimal number of microstates was decided, we ran hierarchical clustering on the average from all the data to define our final microstate set. Every data sample of each group average was then projected onto these microstates to assess the proportion of signal explained by each microstate at each timepoint. To determine when a microstate was significantly engaged, we compared the proportion of variance explained to a null distribution. This null distribution was the variance explained by a random set of topographies (those from each timepoint in the baseline of every subject). If the variance explained by the microstate was significantly greater (right-tailed, *P* < 0.05) than that explained by the random set of topographies, continuously for more than 5% of the postlance period, the microstate was considered active at those timepoints. Note that this could result in multiple microstates being assigned to a single timepoint, more often during periods of transition between 2 microstates. If a microstate appeared multiple times, these occurrences were considered separate events if the separation period was larger than the average duration of that microstate across all occurrences and groups.

Finally, we assessed the differences in the GFP over the 1 second postlance (total field power), and the onset, duration, and power of each microstate between the facial activity groups. These differences were statistically compared with a null distribution that was obtained by generating 2 random groups of surrogate time-series data (as described in Ref. 38), refitting the microstates to the new averages, and extracting the difference parameters (5000 iterations).

## 3. Results

### 3.1. Optimal constellation of facial actions

In both the preterm and term group, there was low occurrence of any pain-related facial activity during the baseline period, with 70.5% of all infants displaying no pain-related facial actions. Of the remaining 29.5%, the majority displayed only open lips, an item previously criticized for its lack of pain specificity,^17,43^ with only 3.8% of the sample showing expressions other than open lips during baseline. There were no sex differences on any of the 3 NFCS constellations in either preterm or term neonates, so no further sex analyses were conducted.

#### 3.1.1. Differential occurrence of discrete facial actions

Brow bulge, eye squeeze, nasolabial furrow, open lips, and horizontal stretch mouth were the most common actions in both age groups with between 46% and 73% infants showing any occurrence (Tables 2 and 3). Examining maximal occurrence (ie, scores of 10/10 on any individual facial action) also showed similar patterns across the 2 groups. Between 17% to 35% of preterms and 8% to 43% of full-term neonates showed a maximal response across the aforementioned 5 actions. Both groups had no maximal response occurrences on vertical stretch mouth and taut tongue, suggesting that these facial actions are unable to pick up the full range of pain-related distress in this cohort.

**Table 2:** Mean scores for the 7 individual pain-related facial actions poststimulus.

	Preterm M (SD); range	Full-term M (SD); range
Brow bulge	5.20 (4.32); 0-10	3.73 (4.21); 0-10
Eye squeeze	5.35 (4.20); 0-10	4.00 (4.33); 0-10
Nasolabial furrow	4.48 (4.29); 0-10	3.42 (4.22); 0-10
Open lips	5.40 (4.57); 0-10	6.46 (4.39); 0-10
Horizontal stretch mouth	4.44 (4.09); 0-10	3.05 (3.87); 0-10
Vertical stretch mouth	1.15 (2.32); 0-9	0.89 (1.97); 0-7
Taut tongue	0.66 (2.08); 0-9	0.24 (0.80); 0-4

**Table 3:** Percentages of pain-related facial actions expressed in preterm and full-term neonates poststimulus.

	Preterm		Full term	
	% showing any occurrence (scores of 1-10)	% showing maximal response (score of 10)	% showing any occurrence (scores of 1-10)	% showing maximal response (score of 10)
Brow bulge	65	22.5	51	19
Eye squeeze	72.5	22.5	51	22
Nasolabial furrow	60	20	47	17
Open lips	65	35	73	43
Horizontal stretch mouth	63	17	46	8
Vertical stretch mouth	27	0	24	0
Taut tongue	10	0	11	0

#### 3.1.2. NFCS-P-3 captured highest total facial action scores

Friedman analysis of variances demonstrated significant differences in the overall scores across the 3 NFCS constellations in both preterm (χ^2^(2) = 32.07, *P* = 0.00) and term (χ^2^(2) = 25.63, *P* = 0.00) neonates (Tables 4 and 5). Post hoc analyses using Wilcoxon signed-rank tests revealed that the NFCS-P-3 score, for both preterm and term neonates, was significantly higher (ie, captured higher behavioural activity related to the heel lance) than both NFCS-7 (linearly scaled to 0-30 for comparison) and NFCS-3 scores.

**Table 4:** Total scores on the 3 Neonatal Facial Coding System variants poststimulus.

	Preterm M (SD); range	Full term M (SD); range
NFCS-7	26.45 (22.54); 0-65	21.90 (20.55); 0-57
NFCS-3	11.08 (9.54); 0-29	7.95 (9.38); 0-27
NFCS-P-3	15.23 (12.48); 0-30	11.29 (12.45); 0-30

NFCS, Neonatal Facial Coding System.

**Table 5:** Within-subject differences between the Neonatal Facial Coding System variants in preterm and full-term neonates poststimulus.

	Preterm		Full-term	
	*Z*	*P*	*Z*	*P*
NFCS-3 vs NFCS-P-3	−4.43	0.00	−3.93	0.00
NFCS-7* vs NFCS-P-3	−4.41	0.00	−2.65	0.01
NFCS-3 vs NFCS-7*	−1.90	0.06	−3.67	0.00

* NFCS-7 total scores were linearly scaled to 0 to 30 for comparison (*M*_preterm_ = 11.62; *M*_full-term_ = 9.38).

NFCS, Neonatal Facial Coding System.

#### 3.1.3. NFCS-P-3 is the optimal constellation of facial actions

**Figure 1.**
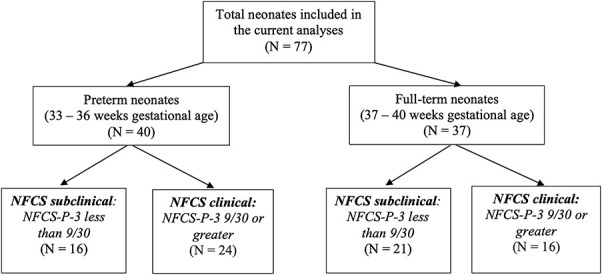
Breakdown of late preterm and full-term subclinical vs clinically significant NFCS groups. NFCS, Neonatal Facial Coding System.

Based on the synthesis of the results above, NFCS-P-3 captured the most variability in both age groups, such that this combination of facial actions occurred the most and resulted in significantly higher pain-related facial activity scores. Based on NFCS-P-3, we grouped preterm and full-term neonates into those expressing clinically significant vs non clinically significant scores (Fig. 1). The distribution that emerged during the poststimulus period, as well as the clinical threshold (9/30) used for our grouping, coincided with the minimal clinically significant pain cut-off score (3/10) in a previous study.^47^ Thus, neonates with scores less than 9/30 were categorized as having no clinically significant pain-related facial activity (NFCS-subclinical), and those with scores equal to or greater than 9/30 were deemed to have clinically significant pain-related facial activity (NFCS-clinical). In both the preterm and term samples, the 2 NFCS groups did not significantly differ for gestational ages, postnatal ages, and sex ratios (Table 6).

**Table 6:** Demographic information of subclinical and clinically significant Neonatal Facial Coding System groups compared in study 2.

	Preterm (N = 40)				Full-term (N = 37)			
	NFCS-subclinical (N = 16)	NFCS-clinical (N = 24)	*t*(38)/χ^2^(1)	*P*	NFCS-subclinical (N = 21)	NFCS-clinical (N = 16)	*t*(35)/χ^2^(1)	*P*
GA (wk)	35.31 (1.10)	35.24 (1.00)	0.22	0.83	38.99 (1.20)	38.80 (1.18)	0.48	0.63
PNA (d)	6.31 (3.95)	5.96 (4.04)	0.27	0.79	5.52 (2.99)	4.38 (2.65)	1.21	0.23
No (%) females	10 (62.5)	13 (54.2)	0.27	0.60	7 (33.3)	5 (31.25)	0.18	0.89

*t* test values provided for gestational age (GA) and postnatal age (PNA); χ^2^ statistics provided for sex ratios.

NFCS, Neonatal Facial Coding System.

#### 3.1.4. Pain-related facial activity did not differ between preterm and term neonates

**Figure 2.**
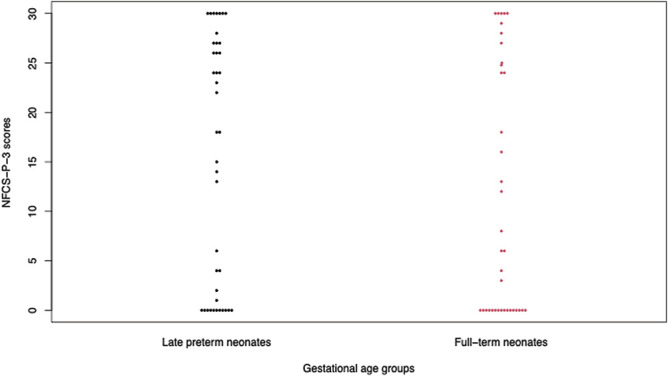
Distribution of poststimulus NFCS-P-3 total scores (range 0-30). Each dot represents a neonate (ie, 2 dots beside a score of 4 in the first column means that 2 preterm neonates out of 40 obtained total NFCS-P-3 scores of 4). The distribution of poststimulus NFCS-P-3 scores did not significantly differ between late preterm and full-term neonates (U = −1.33, *P* = 0.19). NFCS, Neonatal Facial Coding System.

During the baseline period, most neonates did not display any NFCS-P-3 facial actions (97.6% of preterm and 94.6% of term neonates had scores of 0). Considering this lack of variability, baseline pain-related expression scores were not included in subsequent analyses. NFCS-P-3 scores did not significantly differ between the preterm and term groups during baseline (*U* = 0.71, *P* = 0.48) or poststimulus period (*U* = −1.33, *P* = 0.19) (Fig. 2). Both preterm and term groups showed a similar pattern of total scores, with most participants clustering at the extreme ends of the distribution and a minority (approximately 10%) falling in between these 2 clusters (Fig. 2). For the sake of parsimony, only the NFCS-P-3 total score distributions are illustrated in Figure 2 (others available in the supplementary material, available at http://links.lww.com/PAIN/B742).

### 3.2. Pain-related cortical activity is different for preterm and term neonates

**Figure 3.**
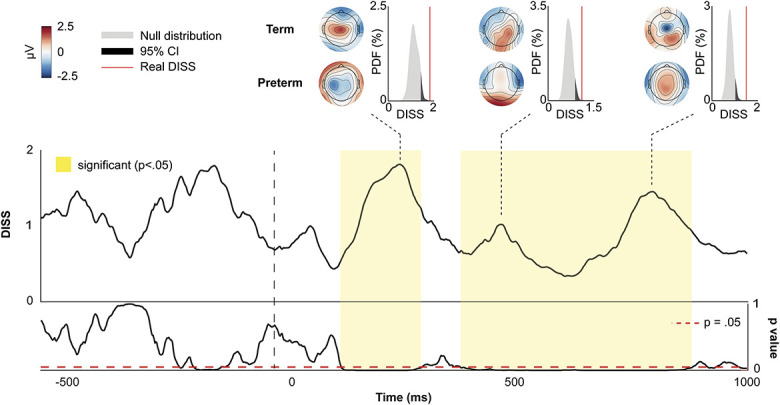
Comparison of cortical activation between term and late preterm neonates. Dissimilarity index (DISS) from comparison of term and late preterm scalp-field topographies during baseline and poststimulus periods (0 represents no difference, whereas 2 represents the polar opposite topography). Bottom panel is the *P*-value of the DISS compared with a null distribution obtained by bootstrapping across groups. Three example timepoints have been selected to demonstrate the 2 group topographies and where the real DISS index for these topographies sits on the null distribution. The dashed vertical line at 0 milliseconds represents the release of the heel lance. PDF, probability density function.

The TANOVA revealed no difference in the topography of the scalp field activity during the baseline period. However, 66.21% of the poststimulus period was significantly different (Fig. 3) between preterm and term neonates.

### 3.3. The overall cortical power postlance is not related to the facial activity score

**Figure 4.**
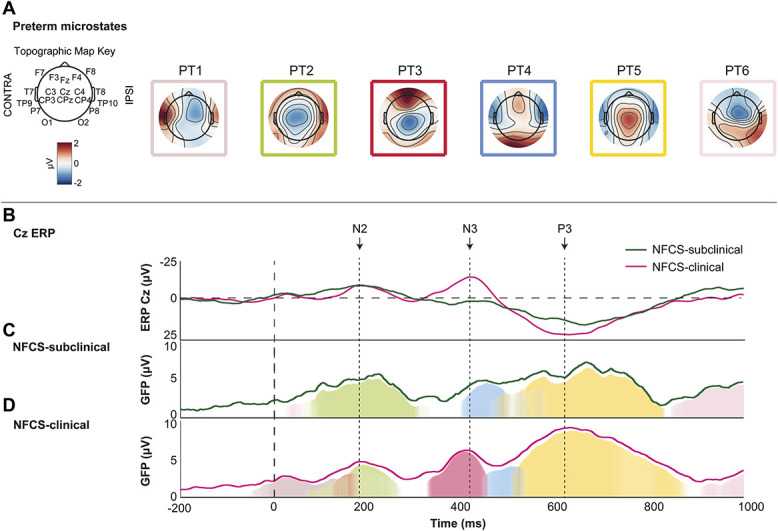
Patterns of microstate engagement in late preterm neonates with subclinical (NFCS-subclinical) vs clinically significant (NFCS-clinical) NFCS scores during the one second after heel lance. (A) Microstates derived from the average across all 40 late preterm participants. (B) Time series comparison of the Cz ERP between the NFCS-subclinical and NFCS-clinical groups. (C and D) Sequence of significant microstate engagement following a clinically significant noxious procedure in the NFCS-subclinical and NFCS-clinical groups, respectively. The dark green and pink lines in C and D, respectively, represent the overall power of the average pain-related cortical activity (GFP) across the whole scalp. The dashed vertical line at 0 millisecond represents the release of the heel lance. ERP, event-related potential; GFP, global field power; NFCS, Neonatal Facial Coding System.

**Figure 5.**
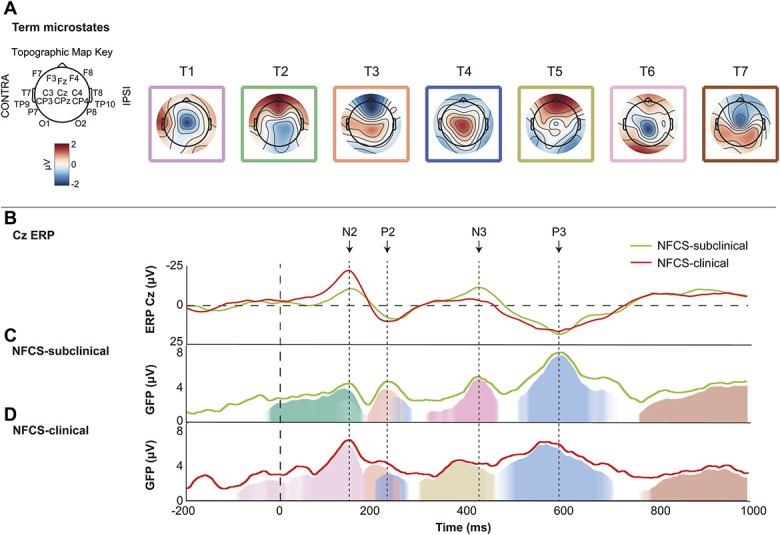
Patterns of microstate engagement in full-term neonates with subclinical (NFCS-subclinical) vs clinically significant (NFCS-clinical) NFCS scores during the 1 second after heel lance. (A) Microstates derived from the average across all 37 term participants. (B) Time series comparison of the Cz ERP between the NFCS-subclinical and NFCS-clinical groups. (C and D) Sequence of significant microstate engagement following a clinically significant noxious procedure in the NFCS-subclinical and NFCS-clinical groups, respectively. The light green and red lines in C and D, respectively, represent the overall power of the average pain-related cortical activity (GFP) across the whole scalp. The dashed vertical line at 0 millisecond represents the release of the heel lance. ERP, event-related potential; GFP, global field power; NFCS, Neonatal Facial Coding System.

Over the first second postlance, preterms displayed a sequence of pain-related cortical activity explained by 6 microstates, which accounted for 84.86% (NFCS-subclinical) and 86.14% (NFCS-clinical) of the cortical signal (Fig. 4). In term neonates, 83.12% of the data in the NFCS-subclinical group and 84.04% in the NFCS-clinical group were explained by 7 microstates (Fig. 5). There was no significant difference in the total power of the average pain-related cortical activity over the 1 second postlance between the 2 NFCS groups for preterm (NFCS-subclinical: M_GFP_ = 3.70 µV; NFCS-clinical: M_GFP_ = 3.98 µV; *P* = 0.12; Fig. 4) or term (NFCS-subclinical: M_GFP_ = 3.35 µV; NFCS-clinical: M_GFP_ = 3.54 µV; *P* = 0.22; Fig. 5) neonates.

### 3.4. Heel lance elicits common sequence of microstates independent of facial activity

In preterm and term neonates, the heel lance evoked the sequential engagement of 4 or 3 (respectively) microstates, which were common to both NFCS groups (Figs. 4 and 5) within each age analysis. In term neonates their degree of activation was not related to facial activity and one (T4—second occurrence) was engaged earlier in the NFCS-clinical group (50.78 ms earlier). In preterm neonates, PT2 was engaged to a lesser degree (229.8 μV × ms less power) and PT5 to a greater degree (358.43 μV × ms more power) in the NFCS-clinical group. PT4 (light blue), PT5 (yellow), and PT6 (pink) were engaged later in the NFCS-clinical group (46.88, 42.97, and 62.5 ms later) (Tables 7 and 8).

**Table 7:** Differences in microstate characteristics between subclinical and clinically significant Neonatal Facial Coding System groups in late preterm neonates poststimulus.

	Onset (ms)			Duration (ms)			Power (ms^2^)		
	NFCS-subclinical	NFCS-clinical	*P*	NFCS-subclinical	NFCS-clinical	*P*	NFCS-subclinical	NFCS-clinical	*P*
PT1*	—	−60.55	—	—	333.98	—	—	425.44	—
PT2	60.55	64.45	0.46	271.48	207.03	0.18	530.39	300.59	0.02†
PT3*‡	—	a: 117.19 b: 320.21	—	—	a: 64.45 b: 136.72	—	—	a: 97.16 b: 328.15	—
PT4	394.53	441.41	0.02†	187.50	99.61	0.01†	382.07	208.33	0.04
PT5	458.98	501.95	0.00†	373.05	382.81	0.34	922.07	1280.50	0.00†
PT6‡§	a: 17.58 b: 839.84	— 902.34	— 0.00†	a: 76.17	—	—	a: 81.05	—	—

Threshold of significance *P* < 0.025 (2-tailed).

* No between-group comparisons were calculated for PT1 and PT3 since they were not engaged in the NFCS-subclinical group.

† Significant results.

‡ Since PT3 and PT6 were visited twice, the properties of the 2 events are provided separately.

§ Although PT6 was activated twice in the NFCS-subclinical group, between-group comparisons were only calculated with the second PT6 event (PT6-b) in the NFCS-subclinical group since it was at a comparable latency with the activation of PT6 in the NFCS-clinical group.

NFCS, Neonatal Facial Coding System.

**Table 8:** Differences in microstate characteristics between subclinical and clinically significant Neonatal Facial Coding System groups in full-term neonates poststimulus.

	Onset (ms)			Duration (ms)			Power (ms^2^)		
	NFCS-subclinical	NFCS-clinical	*P*	NFCS-subclinical	NFCS-clinical	*P*	NFCS-subclinical	NFCS-clinical	*P*
T1	117.19	−97.66	0.01*	68.36	281.25	0.02*	75.15	472.59	0.00*
T2†	−35.16	—	—	212.89	—	—	311.71	—	—
T3	185.55	173.83	0.05	83.98	87.89	0.40	131.49	159.64	0.27
T4‡	a: 210.94 b: 505.86	a: 199.22 b: 455.08	a: 0.12 b: 0.00*	a: 72.27 b: 218.75	a: 76.17 b: 261.72	a: 0.39 b: 0.07	a: 100.58 b: 565.35	a: 109.48 b: 630.62	a: 0.41 b: 0.26
T5†	—	291.02	—	—	171.88	—	—	314.10	—
T6†	310.55	—	—	158.20	—	—	239.64	—	—
T7	765.63	755.86	0.36						

Threshold of significance *P* < 0.025 (2-tailed).

* Significant results.

† No between-group comparisons were calculated for T2, T5, and T6 since these microstates were not engaged in both groups.

‡ Since T4 was visited twice, the properties of the 2 events are provided separately.

NFCS, Neonatal Facial Coding System.

### 3.5. Facial activity is related to distinct microstates during first 500 milliseconds poststimulus

Interspersed with these series of states that were engaged independently from facial activity there were other microstates which were specifically engaged depending on the degree of facial expression changes in both preterm and term neonates (Figs. 4 and 5). In preterm infants, greater facial activity was associated with the additional occurrence of an early microstate (PT1, light brown), which preceded and overlapped with the engagement of the facial activity-independent PT2 state, and a late microstate (PT3, red). In term infants, clinical and subclinical facial activity was associated with a switch in initial (NFCS-subclinical: T2, dark green; NFCS-clinical: T1, purple) and late (NFCS-subclinical: T6, pink; NFCS-clinical: T5, olive) microstates.

## 4. Discussion

This study aimed to determine the relationship between neonatal cortical nociceptive processing and the occurrence of clinically significant pain-related facial expression in preterm and full-term neonates. First, an optimal constellation of facial actions was established corresponding to the facial behaviours from the Premature Infant Pain Profile-Revised for both age groups. After separating infants into clinically significant (NFCS-clinical) and subclinical facial expressions (NFCS-subclinical), based on their poststimulus facial pain scores, we observed a series of microstates reflecting the common sequential activation of pain-related cortical networks, independent of facial expression. Finally, within this common series of activity, the NFCS-clinical groups had additional (preterm) or distinct (term) microstates, suggesting a separate but interweaved sequence of network activation related to the degree of facial expression changes. The results demonstrate that clinical thresholds in pain-related behavioural measures reflect a difference in neonatal nociceptive processing.

### 4.1. Clinical thresholds of pain-related facial expressions reflect changes in nociceptive cortical processing

After a clinically required heel lance, 2 timepoints (during the first 500 ms) were observed at which the premature or term brain engaged different microstates dependent on the expression of clinically significant behaviour. This distinct cortical processing was interleaved with a sequence of 3 or 4 microstates consistent across facial activity groups, reflecting the serial engagement of pain-related cortical processes independent of pain behaviour. Microstates are defined by topography and latency, with each state representing the activation of a single or network of brain region(s). Therefore, a change in microstate reflects (1) an entirely new network or brain region or (2) a change in contribution from different regions within the same network. The results demonstrate that the relationship between pain-related behavioural and cortical responses is more complex than previously believed, as facial activity is related to *how* the brain processes the stimulus, and not simply the degree of activation.^24,27^

The finding of 2 series of activity may reflect the parallel engagement of different pain-processing pathways, only one of which is related to behaviour. This is congruent with the complexity of pain processing, which involves multiple dimensions arising from activation of distinct cortical networks.^3,5,14,33^ The lateral thalamocortical projection pathway projects to the primary somatosensory region (S1) and is associated with the discriminative component of pain,^34,48^ whereas the medial thalamocortical and spinoparabrachial (of the dorsolateral pons) pathways convey information to the anterior cingulate (ACC) and limbic regions, and contribute more to affective processing.^8,35,49,60^ Activation of the spinoparabrachial pathways in rodents leads to autonomic responses and reflexive escape behaviours to avoid further tissue injury, as well as threat learning to avoid future harm.^9,10,30,59^ Indeed, neonatal facial actions originate from the pons and are considered reflexive behaviours to bring about caregiver proximity.^56^ Therefore, the instances of distinct microstates observed here may reflect the differential activation of pathways which are related to reflexive survival behaviours.

Behavioural scales are commonly used to determine if and how much analgesia should be provided; however, not all infants display significant facial activity changes after a noxious procedure.^26,40^ Indeed, within the NFCS-subclinical group, most neonates had no facial response. This suggests that some neonates either do not feel any pain or that they are unable to respond in this way but may display other physiological responses. Neonates with greater physiological stress may be unable to mount a significant behavioural response.^2,27^ This is consistent with the hyporeactive state, described by caregivers and health professionals, observed in some preterm infants due to repeated stress.^16^ This is the first study to demonstrate that there is substantial cortical activity, thus nociceptive processing, despite a subclinical behavioural response. However, clinical thresholds in pain-behaviour are related to changes in specific early components of nociceptive processing. Importantly, those displaying clinically significant pain-related facial expressions are processing the stimulus differently compared with those expressing subclinical facial activity. The degree of cortical activation does not necessarily equate to degree of pain perception, for example, in adults this relationship is not direct.^32^ However, as pain perception ultimately occurs within the cortex, a change is *how* the brain processes a noxious stimulus may have implications for this perception.

### 4.2. The absence of unique microstates in preterm neonates with minimal pain behaviour

Both term groups engaged the same number of microstates at similar latencies; however, preterm neonates with subclinical behaviour had an absence of microstates rather than different microstates to those exhibiting clinically significant behaviour. The absence of a microstate in the group average response does not imply the absence of any cortical activity in individual neonates. Rather, it reflects the absence of consistent activity across most neonates within the group, and only consistent microstates are considered stimulus-related and group-related cortical events.^23^ The absence of consistent activity may, therefore, suggest that these neonates have more variable stimulus-related cortical activity; perhaps, neonates who exhibited minor facial activity changes (a score of 1-8) had different cortical activation to those with no response. Future work will explore the unique brain activity of this group of neonates.

Alternatively, the preterm brain is more immature, with development of functional and structural cortical connectivity occurring throughout the final trimester of gestation. Specifically, connections between regions that process simple stimulus features such as intensity and location develop before those involved in more affective processing.^18,19^ The more immature cortical connectivity may result in preterm neonates failing to engage the behaviour-related networks after insufficient input.

### 4.3. Facial expression related to modulation of common network engagement in preterm neonates only

There was a change in the degree of engagement of common microstates in preterms only. Microstates consistent with the previously described pain-related event-related potentials N2 and P3^20,27,54^ were engaged with lesser and greater power (respectively), in the clinically significant facial activity group. Previous studies have also found a larger P3 in neonates with higher levels of physiological stress^27^ or those held by a parent while clothed (compared with skin-to-skin).^29^ However, these studies involved predominately term neonates and, the microstate between 500 and 800 milliseconds does not reflect the exact same cortical source(s) in term and preterm neonates. Using whole-head microstate analysis, we have shown that the “N2 and P3” states evolve over the final trimester.^55^ Nevertheless, this finding suggests that the degree of activation of “P3” relates to the pain-related facial activity scores in preterm neonates only.

### 4.4. Premature Infant Pain Profile-Revised facial actions capture greatest variability in neonatal facial expression changes

A strength of this work was selection of the optimal constellation of facial actions to determine which babies exhibited clinically significant facial activity. We demonstrated that NFCS-P-3, a coding system based on brow bulge, eye squeeze, and nasolabial furrow, was the only one that captured the full range of pain-related facial activity in both preterm and term neonates. The NFCS-7 and NFCS-3 ranges of total scores did not encompass maximal scores in any infants, although 17.5% of preterm and 13.5% of term neonates showed a NFCS-P-3 maximal response. This finding supports the use of these facial actions in the well-established Premature Infant Pain Profile-Revised^43,45^ for both preterm and term born neonates.

The superiority of NFCS-P-3 in this age group was due to its measurement of facial actions most frequently displayed by neonates. Newborns in the current sample showed patterns of facial actions characterized by frequent brow bulge, eye squeeze, nasolabial furrow, open lips, and horizontal stretch mouth and infrequent displays of vertical stretch mouth (coded by NFCS-7 and NFCS-3) and taut tongue (coded by NFCS-7). Interestingly, preterms and terms showed pain-related facial actions similar to each other but different from older healthy term born infants.^17^ For instance, although the occurrence of vertical stretch mouth carried significant information about pain levels in older term infants,^17^ it is less common in newborns. More effort is involved when engaging vertical stretch mouth compared with other actions^25^; thus, older infants may be better able to mount this action. Taut tongue was also infrequently observed in newborns in past research.^43^ This study bolsters evidence for the unique developmental stage of pain-related facial expressions of neonates (within the first weeks of life).

## 5. Conclusion

This work demonstrated that clinical thresholds in pain-related facial activity are associated with differences in how the brain processes a painful clinical procedure and not simply the degree of brain activity. The neonatal brain engages a series of distinct networks during early stimulus processing, which are related to the magnitude of the immediate behavioural response. Importantly, this distinct activity is interspersed within a series of network activation which is not related to the behavioural response. This complex cortical response observed here may reflect the activation of parallel pain-processing streams with pain behaviours related to only one of these pathways. Although the use of EEG in clinical practice is still far off, this study conducted a fundamental exploration into the relationships between cortical nociceptive processes and clinical thresholds in pain-related facial activity. These results highlight that those neonates who exhibit clinically significant pain behaviours process the noxious stimulus at cortical level differently from those with subclinical scores and that neonatal pain-related behaviours reflect a portion of the overall cortical pain response.

## Conflict of interest statement

The authors have no conflicts of interest to declare.

## Appendix A. Supplemental digital content

Supplemental digital content associated with this article can be found online at http://links.lww.com/PAIN/B742.

## Supplementary Material

SUPPLEMENTARY MATERIAL
