# Supplementary figures and images for: Clinical thresholds in pain-related facial activity linked to differences in cortical network activation in neonates
Source: Pain. 2022 Oct 27;164(5):1039–50. doi: 10.1097/j.pain.0000000000002798 (PMC10108588; doi:10.1097/j.pain.0000000000002798)

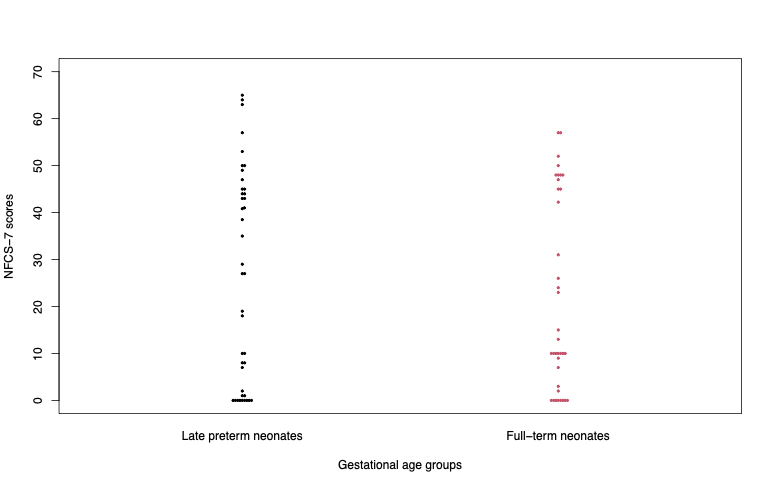

Supplement: SUPPLEMENTARY MATERIAL [file jop-164-1039-s001.tiff]

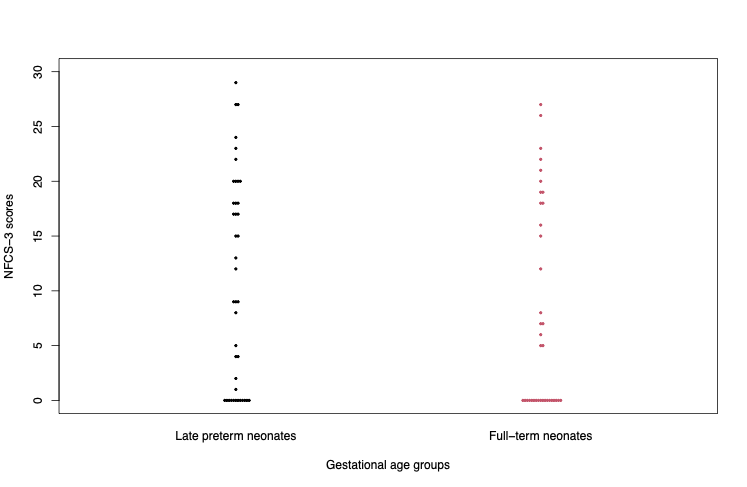

Supplement: SUPPLEMENTARY MATERIAL [file jop-164-1039-s002.tiff]
